# Psychotherapy Is Chaotic—(Not Only) in a Computational World

**DOI:** 10.3389/fpsyg.2017.00379

**Published:** 2017-04-24

**Authors:** Günter K. Schiepek, Kathrin Viol, Wolfgang Aichhorn, Marc-Thorsten Hütt, Katharina Sungler, David Pincus, Helmut J. Schöller

**Affiliations:** ^1^Institute of Synergetics and Psychotherapy Research, Paracelsus Medical UniversitySalzburg, Austria; ^2^Department of Psychology, Ludwig Maximilians UniversityMunich, Germany; ^3^Department of Psychosomatics and Inpatient Psychotherapy, University Hospital of Psychiatry and Psychotherapy, Paracelsus Medical UniversitySalzburg, Austria; ^4^Department of Life Sciences and Chemistry, Jacobs University BremenBremen, Germany; ^5^Department of Psychology, Chapman UniversityOrange, CA, USA

**Keywords:** psychotherapy processes, mathematical modeling, deterministic chaos, common factors, complexity science, psychotherapy integration

## Abstract

**Objective:** The aim of this article is to outline the role of chaotic dynamics in psychotherapy. Besides some empirical findings of chaos at different time scales, the focus is on theoretical modeling of change processes explaining and simulating chaotic dynamics. It will be illustrated how some common factors of psychotherapeutic change and psychological hypotheses on motivation, emotion regulation, and information processing of the client's functioning can be integrated into a comprehensive nonlinear model of human change processes.

**Methods:** The model combines 5 variables (intensity of emotions, problem intensity, motivation to change, insight and new perspectives, therapeutic success) and 4 parameters into a set of 5 coupled nonlinear difference equations. The results of these simulations are presented as time series, as phase space embedding of these time series (i.e., attractors), and as bifurcation diagrams.

**Results:** The model creates chaotic dynamics, phase transition-like phenomena, bi- or multi-stability, and sensibility of the dynamic patterns on parameter drift. These features are predicted by chaos theory and by Synergetics and correspond to empirical findings. The spectrum of these behaviors illustrates the complexity of psychotherapeutic processes.

**Conclusion:** The model contributes to the development of an integrative conceptualization of psychotherapy. It is consistent with the state of scientific knowledge of common factors, as well as other psychological topics, such as: motivation, emotion regulation, and cognitive processing. The role of chaos theory is underpinned, not only in the world of computer simulations, but also in practice. In practice, chaos demands technologies capable of real-time monitoring and reporting on the nonlinear features of the ongoing process (e.g., its stability or instability). Based on this monitoring, a client-centered, continuous, and cooperative process of feedback and control becomes possible. By contrast, restricted predictability and spontaneous changes challenge the usefulness of prescriptive treatment manuals or other predefined programs of psychotherapy.

## Introduction: evidence for deterministic chaos in psychotherapeutic processes

During the past few decades, the conceptualization of psychotherapy as a nonlinear, dynamic, and complex process has been outlined in many publications and by different research groups (Schiepek et al., 1992a, 2014a,b; Orsucci, 2006, 2015; Hayes et al., 2007; Guastello et al., 2009; Pincus, 2009, 2015, 2016; Haken and Schiepek, 2010; Salvatore and Tschacher, 2012; Gelo and Salvatore, 2016). The interest in this approach is increasing, since it is capable of explaining important features of human change processes, including: discontinuous progress (sudden gains or sudden losses, Lutz et al., 2013; Stiles et al., 2003), missing proportionality and nonlinear relations between interventions and outcome (Muran et al., 1995; Hayes et al., 2007; Haken and Schiepek, 2010), unpredictability of long-term courses (Strunk et al., 2015), the dependency of human functioning on specific contexts and situative requirements (Kashdan and Rottenberg, 2010), the eigendynamics and individuality of evolutionary patterns (Barkham et al., 1993; Tschacher et al., 2000; Molenaar, 2004; Fisher, 2015; Fisher and Boswell, 2016), and the important role of client's contributions (e.g., motivation, ressources) to psychotherapeutic gains (Orlinsky et al., 2004; Bohart and Tallman, 2010).

Some authors discuss the nonlinear dynamics approach as a new paradigm or a meta-theoretical framework in psychology (Lichtwarck-Aschoff et al., 2008; Haken and Schiepek, 2010; Orsucci, 2015; Gelo and Salvatore, 2016). We are currently seeing an era where the life sciences, including psychology, become ever more sophisticated and computational in their modeling practices—with high-throughput technologies providing access to different layers of data, from biological to organizational scales, and with simulations becoming an integral part of the discovery process. Driven by the rich data on psychotherapy dynamics obtained with high-frequency feedback (e.g., from the Synergetic Navigation System using standardized questionnaires like the Therapy Process Questionnaire (TPQ), Schiepek et al., 2016a) a quantitative complexity science of psychotherapy processes is now possible. Synergetics, nonlinear dynamics, and the theory of complex systems provide an appropriate theoretical foundation for this endeavor.

Beyond guiding the interpretation of otherwise puzzling empirical and practical matters in psychotherapy, specific conjectures can be deduced from these complexity-based theories. One is the emergence of critical fluctuations which are uniquely predicted by Synergetics. In empirical studies based on daily self-ratings by psychotherapy clients, critical instabilities, or increased fluctuations, could be found just before pattern transitions occurred (Heinzel et al., 2014; Schiepek et al., 2014b), and the intensity of these critical fluctuations was correlated with therapy outcome (Haken and Schiepek, 2010). Furthermore, using critical fluctuations as a marker of order transitions, neuronal activity patterns also changed significantly across these therapeutic transitions (Schiepek et al., 2013).

Perhaps the most crucial, and likewise the most difficult conjecture of the nonlinear dynamics approach, is the emergence of deterministic chaos. Chaos as an umbrella term covers a broad spectrum of irregular and complex system behaviors, which is different from white noise at the one side and from regular oscillations at the other^1^. The phenomenon of chaos is crucial because just the basic assumption of ubiquitous nonlinearly interacting variables implies the possibility of chaotic dynamics. In the case of continuous flow, only three interacting variables are necessary to produce chaotic behavior (Schuster, 1989; Ott, 1993; Strunk and Schiepek, 2006).

Indeed, most biological and mental systems are typically concieved to involve nonlinear relations between multiple components. However, attempts to find empirical proof of chaotic dynamics are ambitious at best, because of the difficulties in finding time series data of sufficient length, scale resolution, and accuracy of measurement. Another major challenge is the ubiquitous transitions that occur within chaotic patterns in adaptive and self-regulatory systems. Psychological and physiological flexibility are fundamental aspects of health (Kashdan and Rottenberg, 2010). With respect to dynamics, this means that healthy systems remain poised to switch attractors depending upon stimulation and demands. These types of chaotic nonstationarities have been observed in default modes in brain functioning (Deco et al., 2013), in chaotic shifts in living systems (Kowalik and Elbert, 1994), and most relevantly within the chaotic phase transitions of learning and psychological development (Haken and Schiepek, 2010). Considering the high likelihood that psychotherapy involves chaotic processes along with the difficulties of identifying it, the empirical validation of the chaos hypothesis in psychotherapy is as important as challenging. The solution to this challenge lies in the use of methods which are sensitive in detecting deterministic chaos, while also able to withstand the presence of nonstationarities in the form of phase transitions.

One early line of research into chaos and dynamic transitions in psychotherapy targeted the dynamics of the therapeutic relationship (Kowalik et al., 1997; Schiepek et al., 1997; Strunk and Schiepek, 2006). The method of these studies was *Sequential Plan Analysis*, which was derived from the *hierarchical plan analysis* approach of Grawe and Caspar (c.f., Caspar, 1996). In this context, “plans” are more or less conscious and verbally or nonverbally communicated intentions and/or self-presentations in a social situation. Using this notion of plans, client and therapist's interactional behavior was analyzed from video recordings of two complete therapies, encoded with a sampling rate of 10 s. At this measurement frequency, a psychotherapy process of 13 sessions was represented by multiple time series of about 3,800 measurement points, and a therapy of 9 sessions by time series of about 2,900 points.

Nonlinearity was proven by surrogate data tests (Rapp et al., 1994) using random surrogates and FFT-based phase-randomized surrogates. The time series were analyzed by methods which are sensitive to the nonlinearity (chaoticity) as well as the nonstationarity of the processes. The estimation of the time-varying change of fractal dimensionality by the method of pointwise correlation dimension D2 (PD2, Skinner et al., 1994) and of the “butterfly effect” of the dynamics by the Local Largest Lyapunov Exponent (LLLE, Rosenstein et al., 1993) was used to identify phase-transition like discontinuities. Following the evolution of the fractal dimensionality by PD2, both therapies displayed nonstationarities, and both therapies showed periods of strongly synchronized and anti-synchronized PD2-processes between client and therapist. Similar, yet even more pronounced dynamical jumps were identified when applying the LLLE, which represents changes in the chaoticity of a time signal (Kowalik et al., 1997). Most of the discontinuities of the LLLE were exactly synchronized between client and therapist. This makes sense in terms of dynamical systems, in that both persons are involved within a self-organizing communication system or relationship, which enables and triggers the individual change process of the client (corresponding to the *generic model* of psychotherapy; Orlinsky, 2009).

These conclusions were supported as well from nonlinear coupling measures between the time series of the interaction partners. Specifically, Pointwise Transinformation and Pointwise Coupling Conditional Divergence (Vandenhouten, 1998) were carried out on the same data, each indicating shifting, time-dependent coupling strengths between the time series of the client and therapist. Interestingly, there was no priority of the therapist's influence on the client, or vice versa. From a systems viewpoint, this circular causality underlying psychotherapeutic self-organization contradicts the classical view that unidirectional input from the therapist determines the client's output.

In sum, these results corroborate the hypothesis of: (i) nonlinearity and deterministic chaos realized in therapeutic change dynamics and interaction, (ii) spontaneous order transitions within these chaotic processes, and (iii) synchronization and synchronized order transitions between client and therapist. Subsequent studies focused on self-organized synchronization between client and therapist at different time scales using an even wider variety of methods (Rockstroh et al., 1997; Ramseyer and Tschacher, 2008; Walter et al., 2010; Gumz et al., 2012).

In another study on ordered dynamics in psychotherapeutic change processes we used the data from daily self-assessments of 149 patients during inpatient psychotherapy (Strunk et al., 2015). The self-ratings were collected by an Internet-based device (the Synergetic Navigation System [SNS], Schiepek et al., 2015, 2016a). Every day, patients completed the Therapy Process Questionnaire (TPQ, inpatient version with 23 items, grouped into 5 subscales) (Schiepek et al., 2012). Most of the patients were categorized into one of three ICD-10 diagnostic groups: F30 (affective disorders), F40 (neurotic stress-related and somatoform disorders), and F60 (specific disorders of personality, esp. F60.3, emotionally unstable personality disorder, referred to as borderline type in other classification systems). On average, the TPQ was completed by patients during 97 days (SD: 50.3). The measurement series of all 149 patients were joined together, resulting in 5 artificial time series (one for each subscale) with a length of *n* = 14,425 points (one time series for each subscale of the TPQ).

The time series of the factors of the TPQ were analyzed by the PD2 algorithm. While D2 provides a complexity estimation (fractal dimensionality) of the attractor of the whole process, PD2 portrays the possible changes of dimensional complexity over time (nonstationarity). D2-estimates are taken from vector point to vector point and can be portrayed in a PD2 × time diagram (Skinner, 1992; Skinner et al., 1994). We adopted Skinner's criterion (Skinner, 1992) of at least 75% valid measurement points for the calculation and interpretation of the PD2. This implies that the majority of the process is suitable for interpretation as ordered dynamics instead of a stochastic process. The arithmetic means of the PD2 measures of the 5 time series ranged from 0.947 to 5.187, indicating a low-dimensional chaotic processes (6 or less independent dimensions). Large standard deviations in the PD2 dynamics were also found, which make sense considering the different levels of fractal dimensionality among different clients, and also to the nonstationarities of the dynamics: order transitions during the course of each treatment.

A crucial aspect of the PD2 analysis is the validation by Fast Fourier Transformed (FFT) surrogate time series. This approach is particularly rigorous and discriminating because it not only contains means and variances of the surrogate time series used for comparison, but also their frequency spectra. Only nonlinear characteristics are removed, providing the basis for determining that there is a statistically significant difference in D2 complexity between empirical and surrogate time series. When nonlinear dynamic structures are destroyed by producing FFT surrogates, one would expect significantly increased fractal complexity of the surrogates. This hypothesis was confirmed: all *t*-tests were highly significant. The hypothesis of chaoticity and nonlinearity of psychotherapeutic processes was corroborated once again.

The identification of chaos in psychotherapeutic change processes may to some seem to be only of academic interest; however the consequences are actually far reaching. First, chaotic processes are sensitively dependent on initial conditions and on small fluctuations, which means that psychotherapy process would be considered to be inherently unpredictable, beyond the bounds of linear control. A second consequence of the chaoticity of change processes is the distinctive individuality of each person's psychotherapy. Any notion of superposition of dynamics within or between individuals (systems) is untenable, meaning that concepts like “standard tracks” or “normative processes” are entirely inappropriate in describing psychotherapeutic change. Since chaotic behavior does not result from irregular input from an *outside* source, but is instead produced by self-organizing processes *within* the system itself, a proof of chaoticity at the same time is a proof of the concept of self-organization. Inherent to this concept of self-organization, there are fundamental doubts about classical notions of input-output mechanisms, such as the role of intervention as a primary force of change, and on strategies aimed at process control by adherence to therapy manuals. By contrast, chaos in psychotherapy processes requires that the therapist remain flexible and attentive to the actual state of the process, particularly concerning its stability or instability over time. Rather than developing more manuals, or selecting this or that technique, psychotherapy may be better supported through the use of real-time process monitoring technologies combined with a continuous collaborative process between therapist and client (Schiepek et al., 2016a).

Beyond the consequences for practical work and empirical research strategies, chaos also brings consequences for the theoretical modeling of change mechanisms. After decades of focusing on the question, *if* psychotherapy works, motivating outcome research, efforts have intensified to understand *how* psychotherapy works (Kazdin, 2005, 2009), taking seriously that the “explanandum” is the change process and that the answer lies within the change process itself, rather than within this or that approach. Theoretical models should be able to simulate the nonlinear dynamics of change processes including all features of deterministic chaos: irregularity of the dynamics, sensitive dependency of the process on initial conditions and on small but well-timed interventions, global stability of the system's behavior within its (more or less stable or transient) attractors, and the dependency of the actually realized attractor on the control parameters of the system, resulting in attractor shifts during the change process. The aim of this paper is to do just that, to demonstrate how a client-cantered, common factors model of psychotherapy can produce each of these features.

## The model

**Figure 1:**
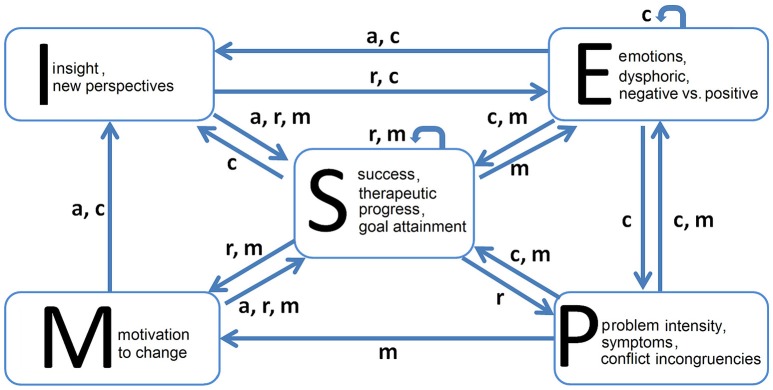
**The structure of the model illustrates the dependencies between the variables and the parameters of the system**.

**Figure 2:**
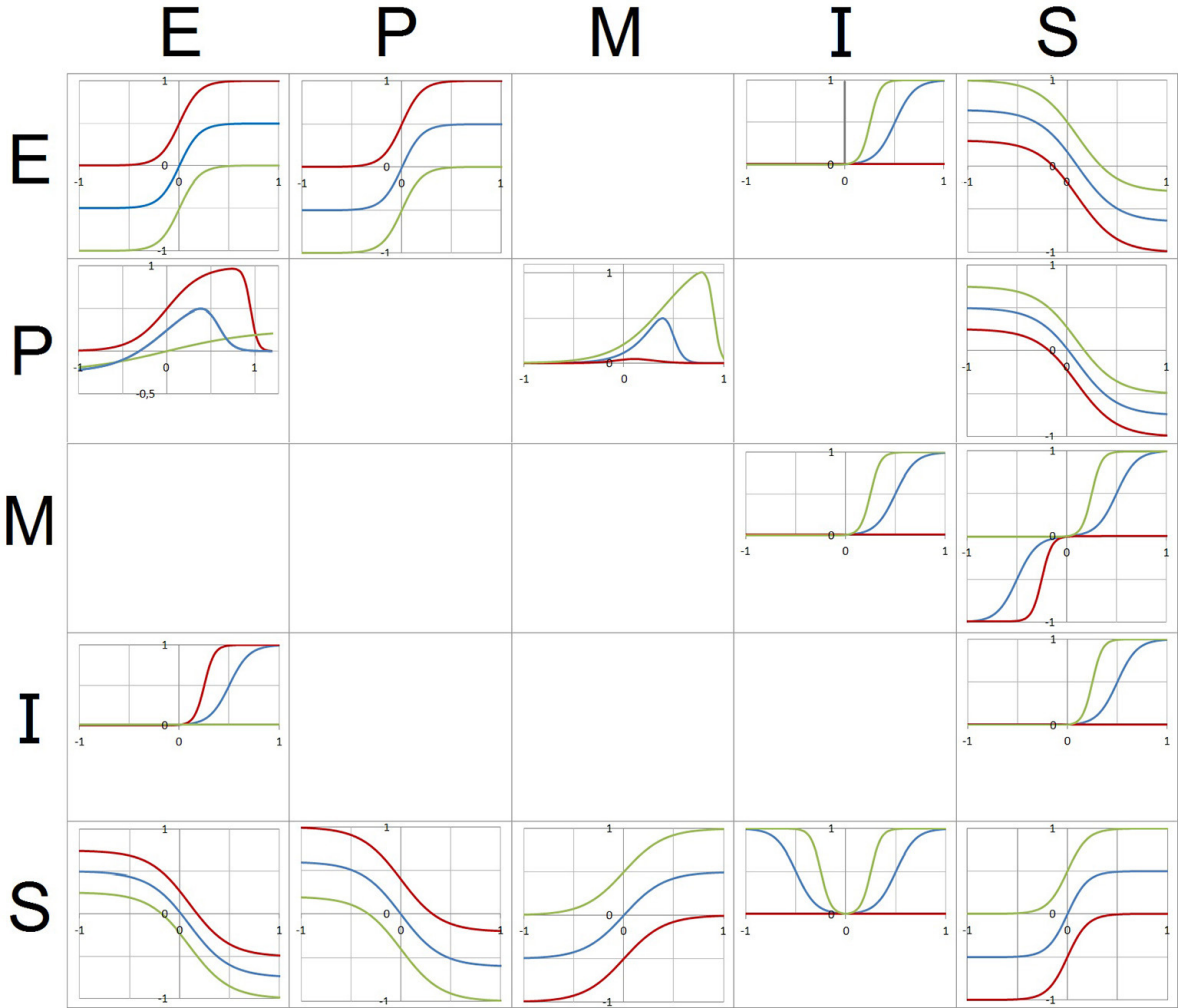
**The figure represents the 16 functions of the model (see text)**. The variables noted at the left side of the matrix (lines) represent the input, the variables noted at the top (columns) represent the output. Each function is represented by a graph in a coordinate system (x-axis: input, y-axis: output). Green function graphs correspond to the maximum of the respective control parameter(s) (= 1), red graphs to the minimum of the parameter(s) (= 0). Blue graphs represent an in-between state (0 < parameter value < 1).

One of the most robust findings in common factors research is the importance of the client contributing to the course and outcome of psychotherapy (Duncan et al., 2004; Orlinsky et al., 2004; Orlinsky, 2009; Bohart and Tallman, 2010; Sparks and Duncan, 2010). For this reason we focus on the variables and the psychological mechanisms which have repeatedly been shown to be important within the “client system” both empirically and theoretically (e.g., Grawe, 2004; Orlinsky et al., 2004). Another reason for choosing these variables is their correspondence to the factors (subscales) of the Therapy Process Questionnaire (TPQ, Haken and Schiepek, 2010), which is used in the routine practice of psychotherapy monitoring. The variables of the model can be seen as psychological states with varying intensities on a given time scale. In terms of Synergetics they represent the order parameters of the system. Here we suppose a sampling rate of once per day, i.e., each iteration of the simulation can be interpreted as a daily measurement of the variables, as assessed by the TPQ. The model is a further development of the model we described in Schiepek et al. (2016b). The differences from the prior model are noted below (paragraph “Functions”). The structure of the model and the interrelations of the variables are shown in Figures 1, 2.

The model focuses on the psychological mechanisms of the client for a couple of key reasons. First, it is well established that any intervention only has an impact if the client reacts on it, what is referred to as “self-relatedness” in the “generic model” of psychotherapy (Orlinsky et al., 2004; Orlinsky, 2009). Another reason—as just mentioned—is the importance of client-related factors to therapeutic effects (Duncan et al., 2004; Bohart and Tallman, 2010). Nevertheless, the model does include other contributions as well, such as: more or less intended interventions; the therapeutic alliance as experienced by the client; daily hassles, or other personal experiences within the client's environment which are represented by punctual or repeated input onto the variables. As a result, the model is not exclusively client-centered. Of course, there are many other contextual impacts on the therapeutic process, such as other patients in an inpatient setting, or impacts from the client's social network(s) in outpatient treatments (see the extended “generic model,” Orlinsky, 2009). But these contextual impacts are not easy to operationalize and their dynamics are not known in detail, and so are difficult to incorporate. Further developments of the model will, however, ideally integrate other systems which are coupled with the client system, such as the therapist's mental functioning as a network of perceptions, emotions, and cognitions with an impact on professional judgment and behavior.

### Variables

The following variables constitute the model:

(E) Emotions. This is a bidimensional variable representing dysphoric emotions at one end of the dimension (e.g., anxiety, grief, shame, guilt, and anger) and positive emotional experiences at the other end (e.g., joy, self-esteem, and flow). This definition of polarity is based upon to the factor analytic results of the TPQ (Haken and Schiepek, 2010).
(P) Problem intensity, symptom severity, experienced conflicts or incongruencies.
(M) Motivation to change, readiness for the engagement in therapy-related activities and experiences.
(I) Insight, getting new perspectives on personal problems, motivations, cognitions, or behaviors (clarification perspective in the sense of Grawe, 2004), confrontation with conflicts, avoided behaviors and cognitions, or with repressed traumatic experiences.
(S) Success, therapeutic progress, goal attainment, confidence in a successful therapy course.

### Parameters

Parameters mediate the interactions between variables. Depending on their values the effect of one variable on another is intensified or reduced, activated or inhibited. Formally they modify the function defining the relationship of two (or more) variables to each other. Psychologically, parameters can be interpreted as traits or dispositions changing at a slower time scale than the variables or states of a system. In terms of Synergetics, the change of control parameters drives the phase transitions of the dynamics (Haken, 2004). The range of the parameters is from 0 to 1. The model includes 4 parameters:

(*a*) Working alliance, capability to enter a trustful cooperation with the therapist, quality of the therapeutic relationship, interpersonal trust. At the one hand, this parameter signifies the disposition to engage in a trustful relationship (attachment disposition) and at the other hand it refers to the realized quality of the therapeutic bond.
(*c*) Cognitive competencies, capacities for mentalization and emotion regulation, mental skills in self-reflection, and the level of structure based upon the Operationalized Psychodynamic Diagnostics (OPD, www.opd-online.net).
(*r*) Behavioral resources or skills which can be applied to problem solving.
(*m*) Motivation to change as a trait, self-efficacy, positive expectations in one's development, reward expectation, and “health plan” as understood through control mastery theory (Weiss, 1993; Silberschatz, 2009).

### Functions

The model covers 16 functions connecting 5 variables (Figure 2). The functions are represented in mathematical terms which are integrated into 5 coupled nonlinear equations (one for each variable, see **Appendix**). The graphs in the coordinate planes (x-axis: input variable, y-axis: output variable) illustrate the dependency of the shape of each function on the parameter values. The development of the model compared to its previous formulation concerns the functions E → E, E → P, I → E, M → S, P → E, S → I, S → M, and S → S.

Beyond the empirical foundation as it is cited in the description of the functions, the model's functions and parameters were supported following an in-progress systematic review of the empirical evidence on common factors (Sungler, 2017). In this review the author compiled the studies underpinning the model and the empirical findings from psychotherapy research and cognitive psychology, motivation psychology, and emotion regulation explaining the psychological mechanisms behind the functions. Where empirical evidence was not available, choices were made following the cited theoretical conceptualizations (e.g., Horowitz, 1987; Mergentaler, 1996; Greenberg, 2002; Grawe, 2004; Silberschatz, 2009). One of the authors (G.S.) is an expert in psychotherapy research and decided on the plausibility of the model assumptions where the available data and findings were not conclusive.

### E → E

Depending on competencies in emotion regulation and mentalization (*c*), emotions can be up- or down-regulated. At low levels of *c* negative emotions like fear, grief, anger, or shame cannot effectively be down-regulated. Stressful emotions are intensified and even moderate positive emotions are transformed into negative qualities. At higher levels of *c* the downregulation of negative emotions can be effectively realized and even moderate negative emotions are transformed into positive ones. *c* plays the role of a bifurcation parameter in the autocatalytic effect of E on itself.

In the previous formulation of the model, the autocatalytic effect only concerned negative emotions, whereas in this actualized function, positive emotions may also be self-activated by positive feedback. The arbitrary threshold at *c* = 0.05 separating the up- or down-regulation of E was eliminated, and the linear function was replaced by a sigmoid growth which implicates a damped effect of E on E at very intensive emotions (instead of unlimited linear growth). Additionally, we introduced an option of transforming moderate positive emotions into negative ones and vice versa, depending on *c*.

### E → I

As outlined also in the I → E function, insight refers to an emotionally “hot” understanding of personally important topics, psychological mechanisms, conflicts, or biographically relevant events, and their impacts on the client's life. In this sense, emotionally important experiences or emotion-associated “states of mind” (Horowitz, 1987) are a condition for such “hot” insights. In terms of Grawe's general psychotherapy model, only activated negative cognitive-affective schemata can produce new qualities of understanding (Grawe, 2004) or an integration of cognitions and emotions with emerging new qualities (“connecting” in the Therapeutic Cycle Model of Mergentaler, 1996). As Greenberg outlined in his emotion-focused approach, the interaction of emotion and self-related cognition (E ⇄ I) is crucial for psychotherapeutic change (Greenberg, 2002). The function E → I is a logistic growth function with an inert onset (small intensities of stressful feelings do not yet activate negative schemata) followed by an exponential increase and finally a damped effect of E on I. It is assumed that mid-size intensities of emotions will be optimal to create emotionally important insight. Overwhelming affects do not fulfill this effect, because they intensify self-protecting defense mechanisms and inhibit learning and self-reflection by neuronal processes (top-down regulation and transmitter dynamics). Mediating parameters are personal competencies in self-reflection and mentalization (*c*) and the quality of the therapeutic alliance (*a*) (only in a safe and appreciative interpersonal relationship may one risk engagement within intensive, self-referential processes, see the “control mastery theory,” Weiss, 1993; Silberschatz, 2009).

Research supports the importance of emotional experiences for cognitive change and for creating problem-related insight by connecting emotions to cognitions (Mergentaler, 1996; Greenberg, 2002; Grawe, 2004). The confrontation with emotional situations and experiences may be one of the core mechanisms in the treatment of affective disorders, whereas avoiding emotions seem to result in negative therapeutic effects (Greenberg and Pascual-Leone, 2006). Conflicts expressed within the therapeutic relationship, such as crisis-repair sequences, or within other social relationships, may facilitate interpersonal learning (Stiles et al., 2014). While emotions seem to be important for self-related processing, arousal and affective intensities beyond a certain level make things out of control and impede learning (Carey et al., 2006). One of the supporting conditions in this process is the therapeutic relationship (parameter *a*) (Weiss, 1993; Silberschatz, 2009; Flückiger et al., 2012), the other is emotion regulation, self-reflection, and competencies in mentalization and self-regulation (parameter *c*) (Bateman and Fonagy, 2013). If clients cannot activate these competencies, interventions are unlikely to be successful (Orlinsky et al., 2004; Dimaggio et al., 2013; Wirtz et al., 2014; Bateman and Fonagy, 2015). There even may be an interaction between *a* and *c*, since the quality of the interpersonal alliance contributes to feelings of control and to reduced fear of overwhelming emotions, and the other way round, this supports emotion-related coping strategies (Sugiura and Sugiura, 2015).

### E → P

The intensity of worrying emotions (E > 0) like fear, anger, grief, or feelings of guilt contributes directly to the experience of problem intensity. In the case of affective or anxiety disorders, such emotions are by definition part of the problem or of the symptoms. The contribution of E to P has the shape of a logistic growth function, with the steepness of the effect depending inversely on *c*: the smaller the capacity in emotion regulation, self-reflection, and mentalization, the more intense the contribution of E to P. If *c* is small, even moderate positive emotions may intensify the experience of problems or strain, and given high levels of *c*, even moderate negative emotions may be converted into reduced problem or symptom intensity. In general, positive feelings like joy and experiences of self-esteem (E < 0) reduce the intensity of problems or conflicts, with the E → P effect depending on the value of the parameter *c*.

Different from the previous model, the linear effect of E on P was replaced by a sigmoid growth function which implicates a sensitive effect of emotions on experienced problem or stress intensity next to the turning point of positive to negative emotions. Like in other modalities of perception, extreme inputs have less impact on perceptual sensitivity than smaller inputs. The unlimited linear growth of the former function was replaced by a more realistic one. Additionally, the function was extended by the possible effect of positive emotions on experienced problem or stress reduction. Depending on *c*, the vertical position of the growth function introduces the option of transforming moderate or—compared with the expectations—insufficient positive emotions into an experience of problems or distress, and conversely transforming moderate negative emotions into successes or stress relievers (i.e., negative values of P).

### E → S

The experience of “negative” emotions like fear, grief, shame, or anger reduces (or is inversely related to) feelings of progress and being successful in solving personal problems. Within a certain range of intensity, the reducing effect on the confidence in a successful therapy course depends on the intensity of worrying emotions. This reducing effect is given by an inverse logistic function with the steepest gradient in a range of mean emotional intensity. Despite this general effect, small to middle degrees of distressing emotions can contribute to an experience of therapeutic progress, since it can be expected that confrontation with personal conflicts, exposure to anxiety-provoking situations or imaginations, and other kinds of focusing on stressful experiences are painful but necessary as a transitional phase in personal development. “Positive” emotions (E < 0) intensify the feeling of being successful and of progressing in therapy. These effects are mediated by parameters *c* and *m*, that is, by competencies in mentalization and emotion regulation, by self-efficacy, and by positive expectations in progress. The less *c* and *m* are available to a client, the more worrying emotions will reduce S.

### I → E

In this conceptualization of psychotherapy dynamics, insight is based on an emotionally “hot” understanding of personally important (in-)congruencies, of conflicts, or of the impact of biographically relevant events (traumata or life events) on the client's mental functioning. Insight is not understood to be abstract or emotionally “cold” knowledge, such as disease-related information as it is communicated within psychoeducation. This holds for true as well if insight refers to new perspectives on possible scenarios of the client's life. Insight (e.g., narrative confrontation and background stories on emotionally important or even traumatic experiences) can activate intense emotions. The activation of emotions doesn't linearly correspond to the personal importance of the insight, but firstly is exponentially increasing with the “intensity” or importance of the insight, followed by a damped effect at higher levels of I. The sigmoid growth function is inversely mediated by *c* and *r*: the less competencies in self-regulation or emotion regulation (*c*) and behavioral skills (*r*) are available, the more insight will trigger powerful or even uncontrollable emotions. In the previous model this function was exponential which in psychological (e.g., perceptual) and biological systems does not correspond to reality.

### I → S

Insights into the background and the psychological mechanisms of a client's problems and the development of perspectives on his/her life will create a feeling of progress in therapy. In other words, understanding is a precondition for progress in problem solving, behavior change, and new qualities of interpersonal relations. The effect of I on S is mediated by a logistic growth function, with the steepness of the gradient depending on *a, m*, and *r*. This effect requires a certain degree of emotional support and safety, given by the therapeutic relationship (*a*), as well as hopeful expectations and trust in personal development (*m*) in order to transform insight into concrete steps of behavior change (S). Of course, skills and behavioral competencies (*r*) are also necessary to transform I to S.

### M → I

In order to create or construct emotionally important new insights, the client has to be sufficiently motivated. The attempt to establish personally meaningful relations between aspects of information may be energy consuming, as does facing of conflicts or emotionally charged memories. Different states of motivation facilitate processes of self-reflection or insight by a logistic growth function, with the steepness of the gradient depending on *a* (quality of the therapeutic alliance supporting the emotionally charged process of self-reflection) and *c* (personal competencies in self-reflection and mentalization).

### M → S

Motivation supports success. With increasing motivation to engage in the therapeutic work, progress becomes more probable. Engagement is an important condition for goal attainment and accomplished steps in problem solving. Additionally, a motivation-related focus on self-efficacy and reward expectation is a prerequisite for any progress to be perceived and valued. The function is a logistic growth function with an inert onset followed by an exponential increase and finally a damped effect of motivation on experienced success. The mediating parameters are *a* (quality of the therapeutic alliance), *m* (reward expectation, self-efficacy), and *r* (personal resources and skills), with the assumption that these conditions help to transform motivational states into therapeutic progress. From the opposite direction, there is an inverted logistic growth function which transforms “negative motivation” into reduced experience of success, failure, or therapeutic loss. “Negative” motivation corresponds to avoidance goals (Grawe, 2004), resistance against change, self-handicapping, self-harm, and failure-oriented motives (Baumeister, 1991, 1993).

Compared to the previous model, this function is symmetrical by combining the growth function of M on S with an inverted sigmoid growth function. This allows the model to take into account the impact of positive *and* negative motivations (“negative” in the sense of resistance, avoidance goals, or failure-oriented motives) with both playing an important role in human change processes. At high levels of *a, r*, and *m* there is no or only a minor effect of “negative” motivation on S, whereas at very low levels of these parameters, “negative” motivation has a more or less negative impact on the experience of S, but no or only a minor positive impact.

### P → E

This function describes a complex relationship between P and E. Increasing problems or conflict intensity activates worrying and distressing emotions. The more severe or stressing the problem, the more such emotions will be triggered (exponential increase). This emotion triggering effect is more pronounced if the person has only minor competencies in emotion-regulation, self-reflection, and mentalization (which are structure functions of the personality in the sense of OPD) (*c*), and reduced expectations in the capacity to solve problems or to manage difficult or stressful situations (self-efficacy expectation, *m*). With higher dispositions or competencies in *c* and *m*, coping strategies for the down-regulation of negative emotions at distinct problem intensities will be available and can be applied. The higher *c* and/or *m*, the lower is the maximum of E and the earlier coping mechanisms and emotion regulation skills will reduce negative emotions. At low levels of *c* and *m*, different degrees of affect intensities cannot be managed or reduced until completely distressing and disturbing emotions (high levels of E) are interrupted, repressed, or disconnected from conscious experience by consuming drugs or alcohol, by self-harm, or by mechanisms of personality dissociation (Nijenhuis and van der Hart, 2011).

This function differs completely from the previous model which simply proposed an inverted U-shaped relation between P and E. The psychological mechanisms behind the function in the present model correspond more closely to findings in emotion regulation (Koole, 2009; Gross, 2015). The prototypic example of this model of emotional dysregulation rests within the psychopathology of Borderline Personality Disorder (BPD), characterized by heightened emotional sensitivity, reactivity, impulsivity, and deficient impulse control, manifesting in behaviors including impulsive aggression and self-harm, triggered by even the most minor of stressors (Lieb et al., 2004; Crowell et al., 2009). The vulnerability to BPD is represented by low levels of *c* and *m*. Hypersensitivity applies to different kinds of stressors, particularly social rejection and interpersonal conflicts (Schmahl et al., 2014). However, research indicates that affective dysregulation is not specific to BPD, but constitutes a transdiagnostic mechanism that manifests in similar ways in different mental disorders (Santangelo et al., 2016). In consequence, the psychology of emotional (dys-)regulation may be a general mediator in the psychological treatment of affective, as well as other classes of disorders.

### P → M

This function describes the dependency of the actual motivation to change on the intensity of problems, conflicts, or symptom severity. It is the suffering or psychological strain component of the broader urge to change something (i.e., avoidance goals in the sense of Grawe, 2004). If there is no problem and no suffering, there is no need to engage in problem solving. With increasing subjective problem intensity, the motivation to change increases exponentially until a maximum level. Beyond this the problem seems too big to be mastered. With the problem intensity exceeding this threshold, feelings of helplessness and expectations of failure will dominate and motivation decreases (compare the findings on “learned helplessness,” Abramson et al., 1978). The degree of the parameter *m* (learned self-efficacy, positive expectations in one's development, reward expectation) defines where in the range of the problem intensity this point of return is reached. The value of *m* defines the way in which problems and strain encourage the actual state of motivation to change (maximum of the function). At high levels of *m* even severe problems encourage activities in problem solving, whereas at low levels of *m* the person feels helpless, discouraged, or paralyzed (depressed mode) even when confronted with minor problems. In this sense small levels of *m* correspond to the construct of “hopelessness” (Beck et al., 1993).

There is a wide range of empirical evidence on the different aspects of the P → M function, especially concerning the moderating effect of *m*. Some studies show that problem and symptom intensity increase the motivation to change and activate the search for and the utilization of health care providers (Ryan et al., 1995; Rapp et al., 2003; van Beek and Verheul, 2008). Motivation to change is given at higher levels of self-efficay (*m*) even in patients with severe problems like substance abuse and various comorbidities (Schmidt et al., 2009). At low levels of self-efficacy and low self-regulation competence, activities seem to be blocked and persons are more dependent on external motivation (Derryberry and Reed, 1994), with a gap between intention/motivation and action (e.g., procrastination; Steel, 2007) along with low motivation for health-related activities (Sirois, 2004). High levels of intrinsic motivation and self-efficacy contribute to the application of coping strategies during psychotherapy (Caviness et al., 2013) as well as to health-related behavior (Conner and Norman, 1995).

### P → S

Problem intensity has a negative impact on experienced success. If problems, symptoms, or conflicts increase (P > 0), the perceived success and progress is reduced. From the opposite direction: a decrease in problems or symptoms (P < 0) will be perceived as success. The function is an inverse logistic function with the steepest effect gradient of P on S in the vicinity of P = 0. Problems and symptoms have a higher negative impact on S if the parameters *c* (cognitive competencies, e.g., in self-regulation and emotion control) and *m* (reward and self-efficacy expectations) are low, and they have less reducing impact on S if *c* and *m* are high. Persons with more distinguished cognitive competencies and learned self-efficacy are more resilient and robust against problem exacerbations, relapses, or personal crises. In the other direction, problem solving (P < 0) is experienced as personal success.

### S → E

Experiences of success and therapeutic progress reduce the intensity of negative emotions and intensify positive emotions and self-esteem. This reducing effect is given by an inverse logistic function with the steepest gradient in a middle range of success. Conversely, failures or reduced therapeutic progress (S < 0) intensify bad feelings. This effect is mediated by *m*, that is by self-efficacy, positive expectations in the therapeutic progress, or “trait” motivation. The more pronounced the parameter *m*, the better success and therapeutic progress will activate positive emotions and self-esteem, and the less failures or setbacks will activate worrying emotions.

### S → I

Increases in therapeutic success or progress produce information on how problems can be solved. One aspect is the motivating effect of success (S → M) with motivation facilitating the examination of and the involvement in personal topics (M → I). Another aspect is information created by therapeutic progress. This is based on some kind of quasi-experimental relation between changed behavior (independent variable) and its effect on mental functioning, behavior, or social experiences (dependent variable). Success produces insight in the sense of information. The same is true for failure. Just as in a scientific experiment, the rejection of an hypothesis also creates information. In consequence S → I is a symmetric logistic growth function with an inert onset followed by an exponential increase and finally a damped effect of S on I. The symmetry of this function is different than its previous formulation, which only considered the positive branch of S (S > 0). As far as cognitive processes (information processing, mentalization, observation and reflection of one's behavior in relation to the effects on the behavior of others or oneself) are important, the parameter *c* plays a crucial role in shaping this function. Its steepness depends on the value of *c*.

### S → M

Success motivates. With therapeutic progress and growing confidence in a successful therapy, the motivation to engage in the therapeutic work increases. The effect of therapeutic success and reward experiences on motivation follows a logistic growth function with an inert onset (small successful steps at first do not yet trigger big jumps in motivation), followed by an exponential increase, and finally to a damped effect of success on motivation. The parameters *r* and *m* determine the magnitude and steepness of the motivation gradient in the growth function. The more the client can trust his/her behavioral skills or resources, self-efficacy, and reward expectations, the more motivation will play a beneficial role. Low resources and low self-regard together with the expectation of failure reduce motivation. This is not only true in the case of experienced failure and therapeutic losses (i.e., negative success), but also for small degrees of success which in a depressed attitude frame are not sufficient to be experienced as positive. The point symmetry of this function is different from its previous formulation, which only considered the motivating effect of success, and did not include the disencouraging effect of failure or of unsufficient success below the threshold of expectation. Each may either support or impede the therapeutic progress.

### S → P

Problem intensity is reduced by increasing therapeutic success and experienced progress. Positive experiences during psychotherapy (e.g., positive intra-session outcome) and steps onto a desired goal have a reducing impact on demoralization or emotional problems, and thereby reduce the self-perceived problems of a client. The effect is represented by an inverse logistic growth function with the steepest effect gradient of S on P in the vicinity of S = 0. S > 0 reduces P, S < 0 increases P. The effect is mediated by *r*, that is, by the behavioral resources and skills a person can apply to the transformation of new and positive experiences made in therapeutic situations into problem solving and problem reduction in everyday situations. Just as in the other functions (e.g., S → M, S → E, E → P), there is an effect range of S on P in the vicinity of S = 0 which represents a more depressive or a more optimistic frame of attitude.

### S → S

Success enhances and facilitates success, and the other way round, failure and therapeutic losses reduce the experience of success. The intensity of this autocatalytic effect of S depends on *m* (trait motivation, self-efficacy, and reward expectation) and *r* (resources and skills).

In the previous formulation of the model, the autocatalytic effect only referred to positive success, whereas in this newer actualized function the effects of failures and setbacks are represented. Disappointments can be catalyzed as well by downward-oriented “positive” feedback. The option of transforming moderate (sub-expectation) success into disappointment and of minor failures into feelings of success (depending on *m*) also was introduced.

The mathematical terms representing these functions are integrated into 5 coupled nonlinear difference equations. Each equation describes the development of a variable, depending on other variables, on itself, and on the involved parameters (see **Appendix**).

$$\begin{array}{l} E_{t}={\text{f}}_{1}\left(E_{t},I_{t},P_{t},S_{t},\text{c},\text{r},\text{m}\right)\\ P_{t}={\text{f}}_{2}\left(E_{t},S_{t},\text{c},\text{r}\right)\\ M_{t}={\text{f}}_{3}\left(P_{t},S_{t},\text{r},\text{m}\right)\\ I_{t}={\text{f}}_{4}\left(E_{t},M_{t},S_{t},\text{a,c}\right)\\ S_{t}={\text{f}}_{5}\left(E_{t},I_{t},M_{t},P_{t},S_{t},\text{a,c},\text{r},\text{m}\right) \end{array}$$

The system was programmed in Excel 2007 and for reasons of validation also in Matlab (Matlab R2016a Ver. 9.0.0.341360, 64 Bit, www.mathworks.com). In this paper we focus on the deterministic functioning of the network dynamics which corresponds to the concept of deterministic chaos (Schuster, 1989). Further steps toward a more realistic simulation of a specific client would have to consider the trait or parameter dynamics, dynamic and measurement noise, and an empirical input function representing the therapeutic interventions onto the system (see Discussion).

## Results

**Figure 3:**
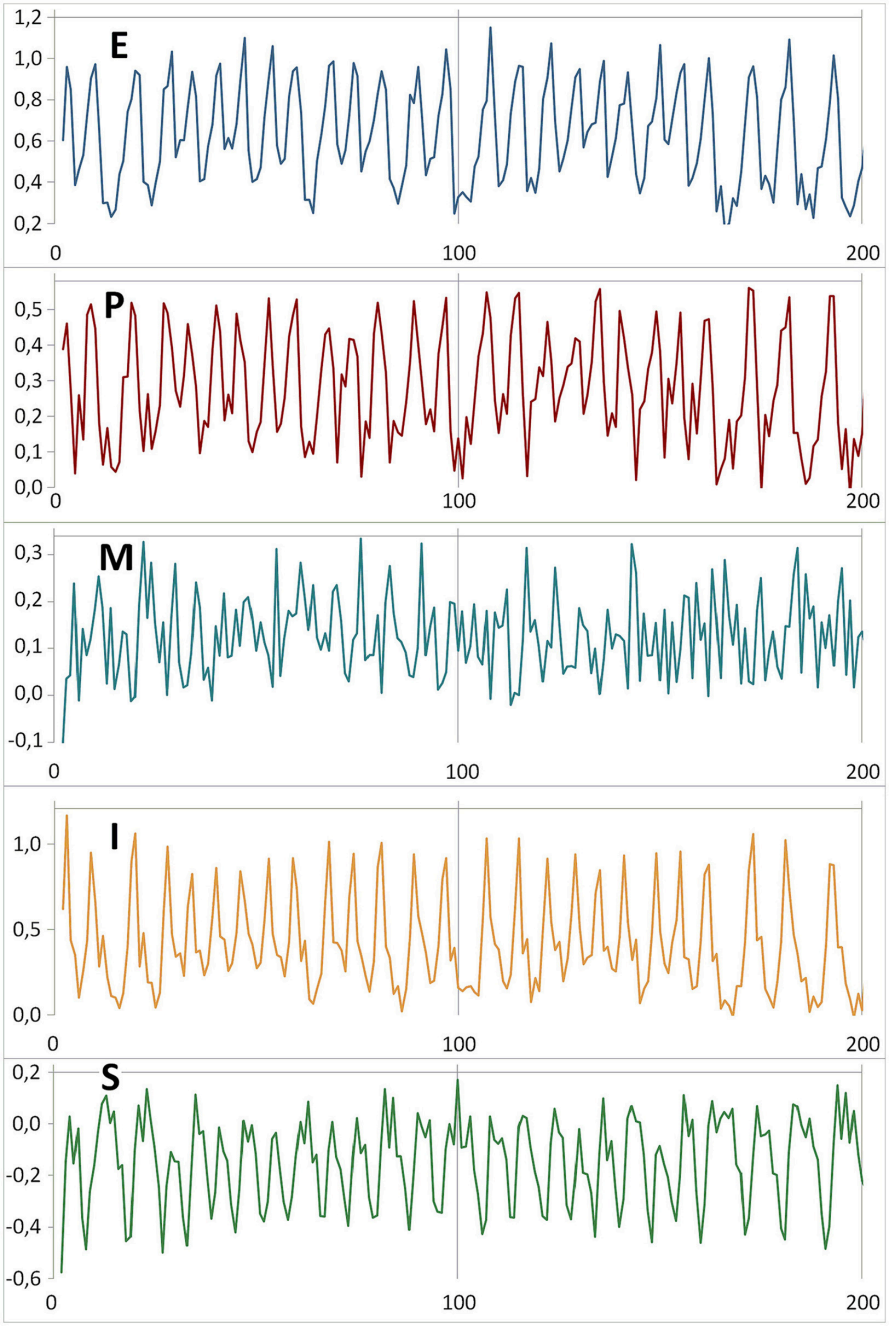
**Chaotic dynamics of the variables E, P, M, I, S**. The initial conditions (values at *t* = 0) are E: 0.99, P: 0.57, M: −0.34, I: 0.01, S: −0.32. Here the time series from the first iteration at *t* = 1 until *t* = 200 are shown. The parameter values of this simulation run are *a*: 0.400, *c*: 0.675, *r*: 0.740, *m*: 0.475.

**Figure 4:**
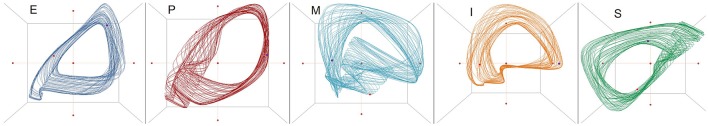
**The attractors of the variables E, P, M, I, S in a chaotic regime with parameters and initial conditions as in Figure 3: *a*: 0.400, *c*: 0.675, *r*: 0.740, *m*: 0.475; E: 0.99, P: 0.57, M: −0.34, I: 0.01, S: −0.32**. Three-dimensional time delay embedding with τ = 1. The attractors are based on 413 valid iterations (the last iterations from a simulation run of 5,000 iterations) splined by the Excel standard spline function.

The model can be seen as a repository of a large amount of empirical information and knowledge about psychotherapy. In the following results, we will show that this representation of a psychotherapy system is capable of generating plausible time series for the dynamical variables, and of displaying many of the complex phenomena associated with temporal process of psychotherapy (e.g., bi- or multistability and transitions related to interventions). In particular, the model is capable of chaotic dynamics. Figure 3 illustrates an example of the irregular (chaotic) dynamics of the variables E, P, M, I, and S. The time-delay embedding of the time series shows the characteristic picture of strange or chaotic attractors (Figure 4). The impression is that of complex but ordered processes, with trajectories following the shape (Gestalt) of the attractor. Within this shape there is an exponential divergence of closely adjacent trajectories but also a convergent trajectory stream which keeps the dynamics within the attractor. The general impression of parallel trajectory pathways mirrors the deterministic generative mechanism of chaos which is quite different from noise or randomness (Kaplan and Glass, 1992).

**Figure 5:**
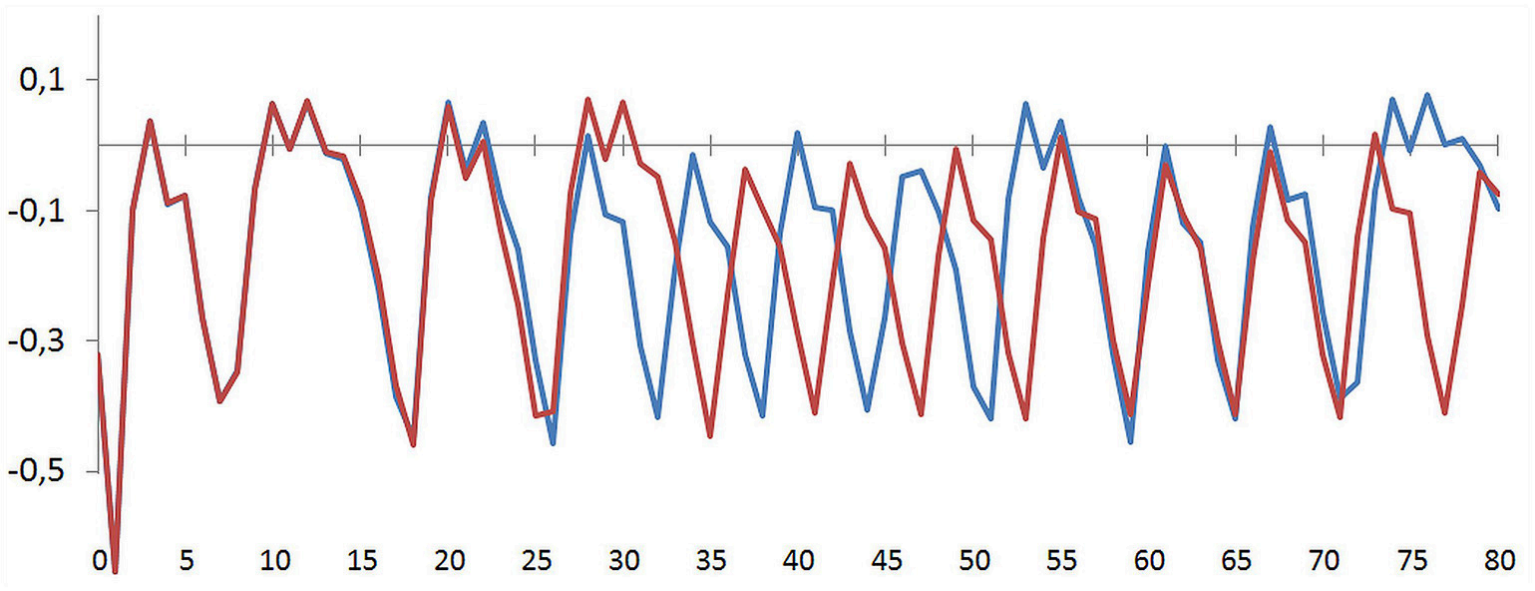
**Sensitive dependency of the dynamics on its initial conditions (variable S, 75 iterations of the simulation run)**. S starts at −0.3200 (blue line) and in a second realization at −0.3201 (red line). Initial values of the other variables (here not shown) as in Figure 3: E: 0.99, P: 0.57, M: −0.34, I: 0.01. Both simulation runs are realized at this parameter values: *a*: 0.400, *c*: 0.675, *r*: 0.740, *m*: 0.475. Even if after some cycles the dynamics of two separately started realizations reapproach, the dynamics follow their own and different ways within the global shape of the existing attractor.

One of the most prominent features of a chaotic processes is its sensitive dependency on initial conditions, with the potential for large differences over time arising from small minor fluctuations within the system, or via inputs from the outside. This so called “butterfly effect” is the reason why the principle of “strong causality” (similar causes have similar effects) does not hold for chaotic systems and also why any long-term prediction of such systems is impossible (see Figure 5 for a realization of the variable S). The prediction horizon depends on the value of the system's Largest Lyapunov Exponent (LLE) (Schuster, 1989; Ott, 1993). The LLE of a time series is a measure of the exponential divergence of trajectories starting nearby in a phase space. The LLEs of the dynamics of E, P, M, I, and S as shown in Figure 4 were calculated by the algorithm of Rosenstein et al. (1993) using 5,000 iterations (parameter values and initial conditions as in Figure 4) and an embedding dimension of 5. The LLEs are: E: 0.007 (τ = 31), P: 0.008 (τ = 17), M: 0.219 (τ = 1), I: 0.225 (τ = 1), S: 0.005 (τ = 24). All LLEs are > 0, indicating chaotic dynamics.

**Figure 6:**
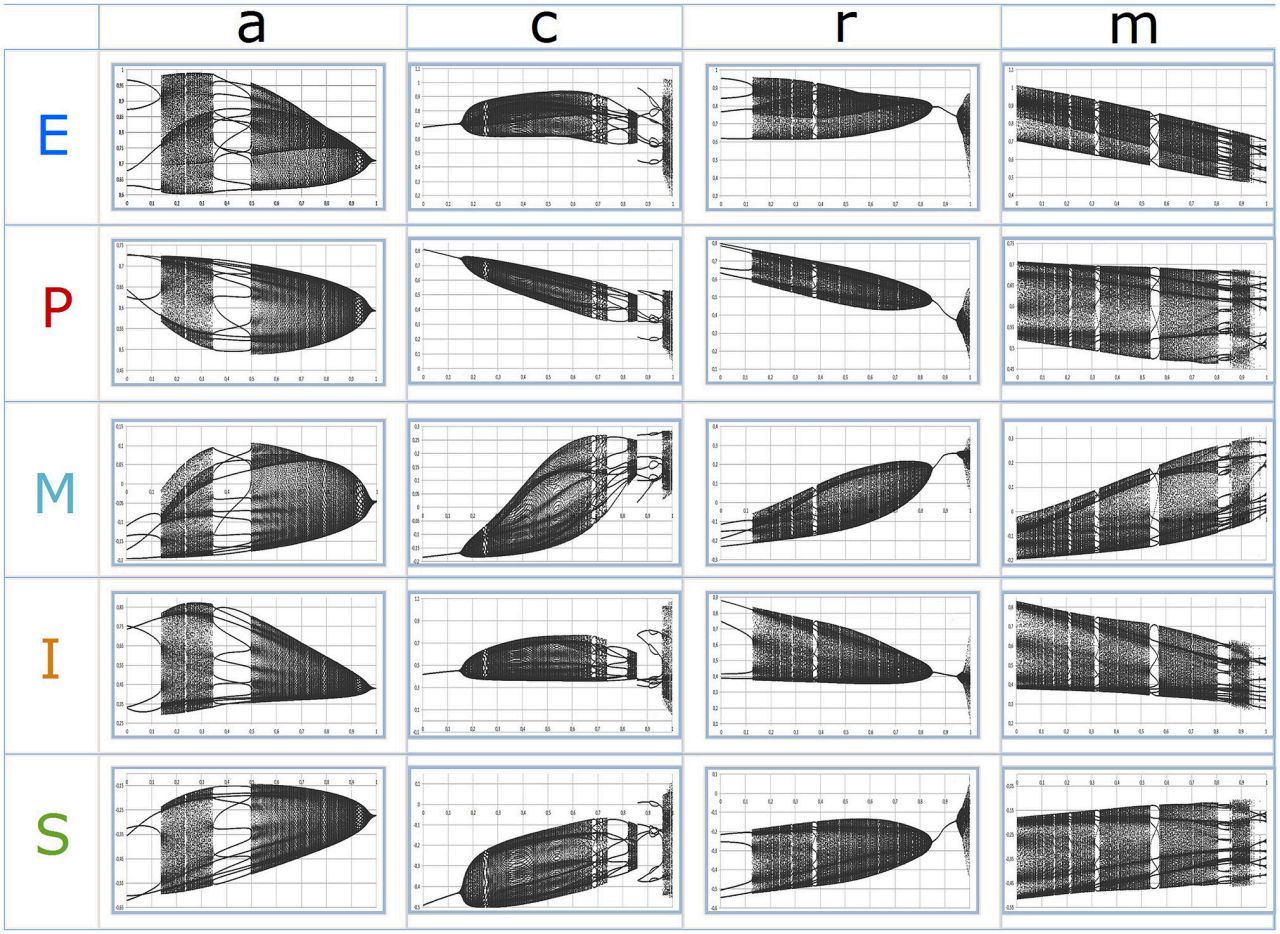
**Bifurcation diagrams**. The x-axis of each diagram represents increasing parameter values, the y-axis the realized values of the variables at each parameter value. The first 200 iterations (transient dynamics on the way to a stable attractor) were removed, the following 250 iterations (number of values at the y-axis) are taken to create the vertical distribution of values at a certain point of the x-axis. The initial conditions of the variables are for all simulation runs: E: 0.99, P: 0.57, M: −0.34, I: 0.01, S: −0.32. The shown parameter range of each diagram is restricted to a certain window: *a*: 0.05–0.90; *c*: 0.35–0.71; *r*: 0.40–0.78; *m*: 0.50–0.65. Within the range, 500 steps of increasing parameter values are shown. The parameters which were not stepwise increased were kept constant at *a*: 0.55065; *c*: 0.50012; *r*: 0.55010; *m*: 0.55100. Outside of the shown parameter range the dynamics is characterized by fixed point or oscillating behavior.

As it is known from other model systems (e.g., the Feigenbaum scenario of the Verhulst map, May, 1976; Schuster, 1989) nonlinear systems do not always behave chaotically, but instead realize a spectrum of fix point dynamics, simple or more complex oscillations, and chaos of different degrees of complexity, depending on the respective parameter values. Our network model covers this spectrum of behaviors. This is visualized by bifurcation diagrams where the long-term behavior of a system is plotted against the parameter value which was used to create the dynamics (Figure 6). The realized states are plotted at the y-axis and the realized parameter values at the x-axis. The most interesting part of the system behavior are complex oscillations and chaos, which realizes many or, in a strict sense, an infinite number of states. The bifurcation diagrams illustrate that the chaotic regime which is realized at certain ranges of the corresponding parameters is interrupted by windows of regularly oscillating patterns. This illustrates the fact that chaos not only sensitively depends on initial conditions or microfluctuations, but also on parameter values.

The overall trends in the mean values of the variables E, P, M, I, and S demonstrate the plausibility of the parameter effects on the variables. If we take the mean of all realized values of a variable at a certain parameter value and correlate it with the parameter intensity of *a, c, r*, and *m*, all parameters are negatively correlated with problem intensity (P) and positively correlated with motivation to change (M) and therapeutic success (S). Following the interpretation of *a, c, r*, and *m* as cognitive, emotional, and behavioral competencies, this pattern of correlations means that more competent clients produce better outcomes. Cognitive competencies (*c*) correlate positively with insight (I), but not *a, r*, and *m*. E is negatively correlated with *a* and *m* (positive emotions and reduced “negative” emotions correspond to higher levels of working alliance and trait motivation), but E is positively correlated with *c* and *r* (which at first glance may seem to be counterintuitive) (Table 1).

**Table 1:** **The arithmetic mean of the dynamics of a variable at a certain parameter value is correlated with the respective parameter values of *a, c, r*, and *m***.

	***a***	***c***	***r***	***m***
E	−0.994	0.587	0.721	−0.893
P	−0.994	−0.999	−0.999	−0.734
M	0.994	0.992	0.997	0.874
I	−0.998	0.884	−0.982	−0.249
S	0.973	0.068	0.987	0.791

*The parameter values were increased by steps of 0.01 from 0 to 1 (= 100 steps). For calculating the variables at each parameter step, the first 100 iterations representing the transient dynamics to the stable attractor were removed, and 250 iterations were taken for calculating the mean of the respective variable at each parameter step. Over all, higher competencies correlate with higher values of M and S, and lower values of P*.

**Figure 7:**
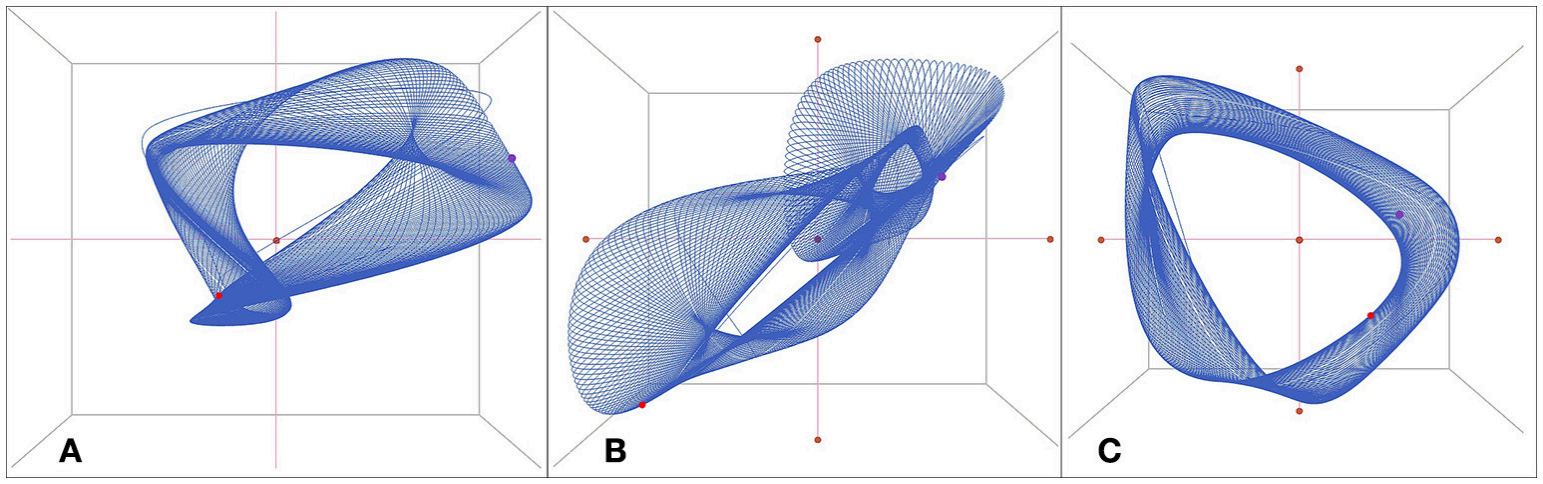
**Attractors based on complex but regularly oscillating time series**. The attractors are realized at the following parameter values: *a*: 0.400, *c*: 0.477, *r*: 0.708, *m*: 0.503. The first 136 out of 450 iterations representing the transient dynamics to the stable attractor were removed. The attractors are reconstructed by the following 314 valid iterations splined by the Excel standard spline function. **(A)**: E, M, and I embedded in a 3-dimensional phase space without time-delay. **(B)**: E, embedded in a 3-dimensional phase space defined by time-delay coordinates, τ = 4. **(C)**: M, embedded in a 3-dimensional phase space defined by time-delay coordinates, τ = 3.

As stated above, the system realizes not only chaotic, but also fix point and oscillating behavior. Figure 7 illustrates attractors representing complex regular oscillations, embedded in a 3-dimensional phase space defined by E, M, and I (without time delay, Figure 7A), by E (time-delay coordinates, τ = 4, Figure 7B), and by M (time-delay coordinates, τ = 3, Figure 7C). The regular structure of the trajectories represents the recurrent oscillations of the time series.

**Figure 8:**
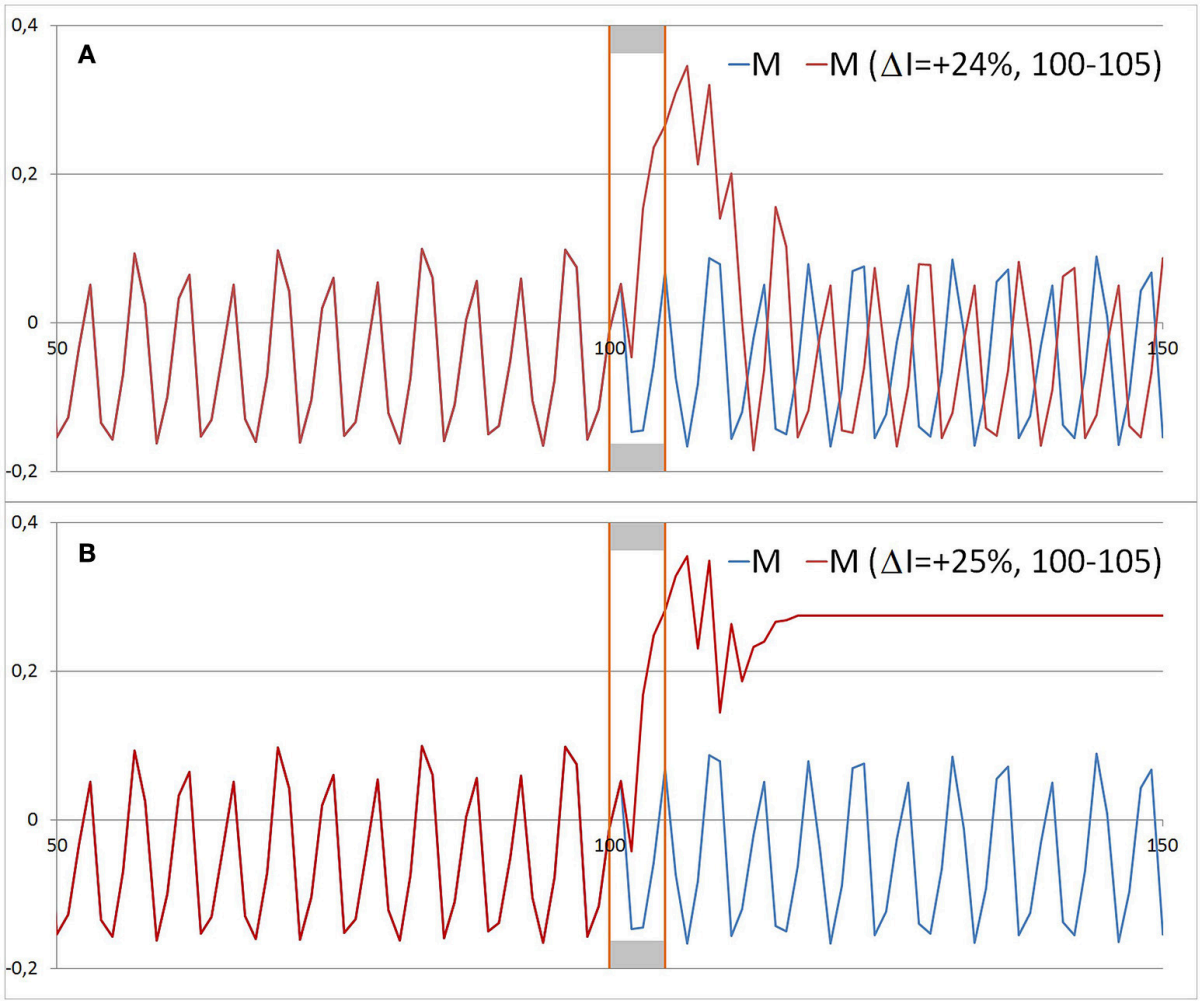
**Small differences in the intensity of interventions on I produce changed dynamics in M**. Same initial conditions and parametrization as in Figure 3. **(A)** From iteration 100–105 an intervention of + 24% on I was realized. After a short period of iterations, a similar but not identical dynamics of M as before was realized. The dynamics runs within the same attractor, but not on the identical trajectory (“butterfly effect“). **(B)** Only a slightly increased intervention strength on I (+ 25% instead of 24%) turns the dynamics of M into a fix point attractor.

**Figure 9:**
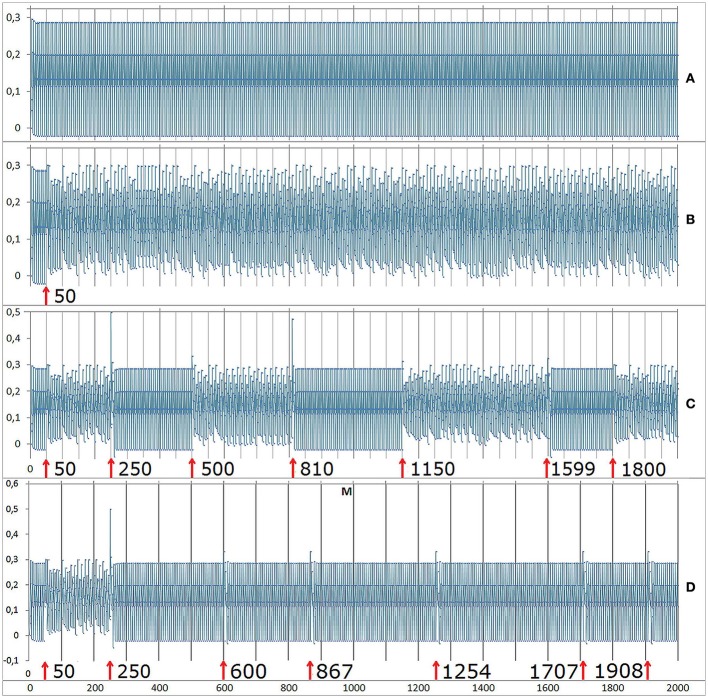
**Time-dependent effects of interventions onto the system at initial conditions of E: 0.99, P: 0.57, M: -0.34, I: 0.01, S:** −**0.32, and parameter values of *a*: 0.05**, ***c*****: 0.71**, ***r*****: 0.78**, ***m*****: 0.65**. Here the time series of M is shown. **(A)** Without interventions, M oscillates regularly. **(B)** An intervention of 20% at *t* = 50 shifts the dynamics from a regular oscillation to a chaotic regime. **(C)** Interventions (20%) at certain time steps produce instantaneous shifts between chaotic and regular oscillations. **(D)** Interventions (20%) at other time steps create only very short deviations from regularity. The oscillatory attractor is reestablished after some few iterations.

The complexity of the dynamics not only appears in the chaoticity of the system, but also in its sensitivity to specific interventions. In the range of stable behavior, most interventions onto the system have no impact on its long-term behavior, and the existing attractor will be reestablished after the displacement (Figure 8A). However, in the range of instability, a small increase of the intervention strength can trigger the system into a quite different attractor (e.g., from a chaotic to a fix-point attractor, see Figure 8B). In this case, an indirect intervention was realized (on I with impact on M). At the edge of instability, interesting phenomena occur (Figure 9): a small input triggers the dynamics into another type of dynamics, and by a second input, the activated dynamics can be switched off (e.g., from complex regularity to chaos and back to regular oscillations). Given specific parameter values, it seems possible to switch the dynamic patterns on and off, but only at appropriate moments. This corresponds to the well-known “kairos” phenomenon of sensitive time windows for decisions or actions. Outside of these sensitive moments, similar interventions have no switching effects. The switching effect is a proof of the bi- or multistability of the system. This means that the system is able to create two or more dynamic patterns at the same set of parameter values. Depending on the initial conditions of the process, on a specific input, or even on small fluctuations, the system will manifest one of the different potentially available patterns.

**Figure 10:**
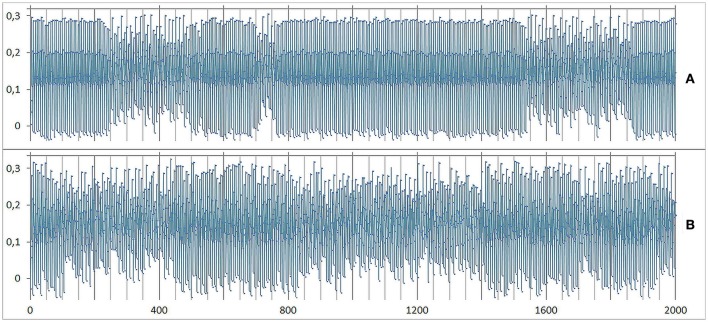
**Dynamic noise on M. Same initial conditions and parametrization as in Figure 9**. **(A)** At a small level of noise (2%) the shifting pattern between regular oscillations and chaos emerges spontaneously. **(B)** At a noise level of 6% the shifting pattern disappeared or at least is completely smeared.

Up to now, we referred to deterministic dynamics without considering any dynamic noise onto the system behavior. Dynamic noise means that noise from the outside or from the inside of a system is processed by the mechanisms of the system (other than measurement noise, which has no impact on further iteration steps, see Hütt, 2001). Dynamic noise is like continuous erratic interventions onto the system. Indeed, small degrees of dynamic noise create the mentioned switching effect, e.g., between irregular (chaotic) and regular dynamics, as it was created by specific time-sensitive interventions (Figure 10A, compare with Figure 9C), whereas higher degrees of dynamic noise blur this effect. As shown in Figure 10B, a switching between different dynamic patterns can only be presaged in the time series.

**Figure 11:**
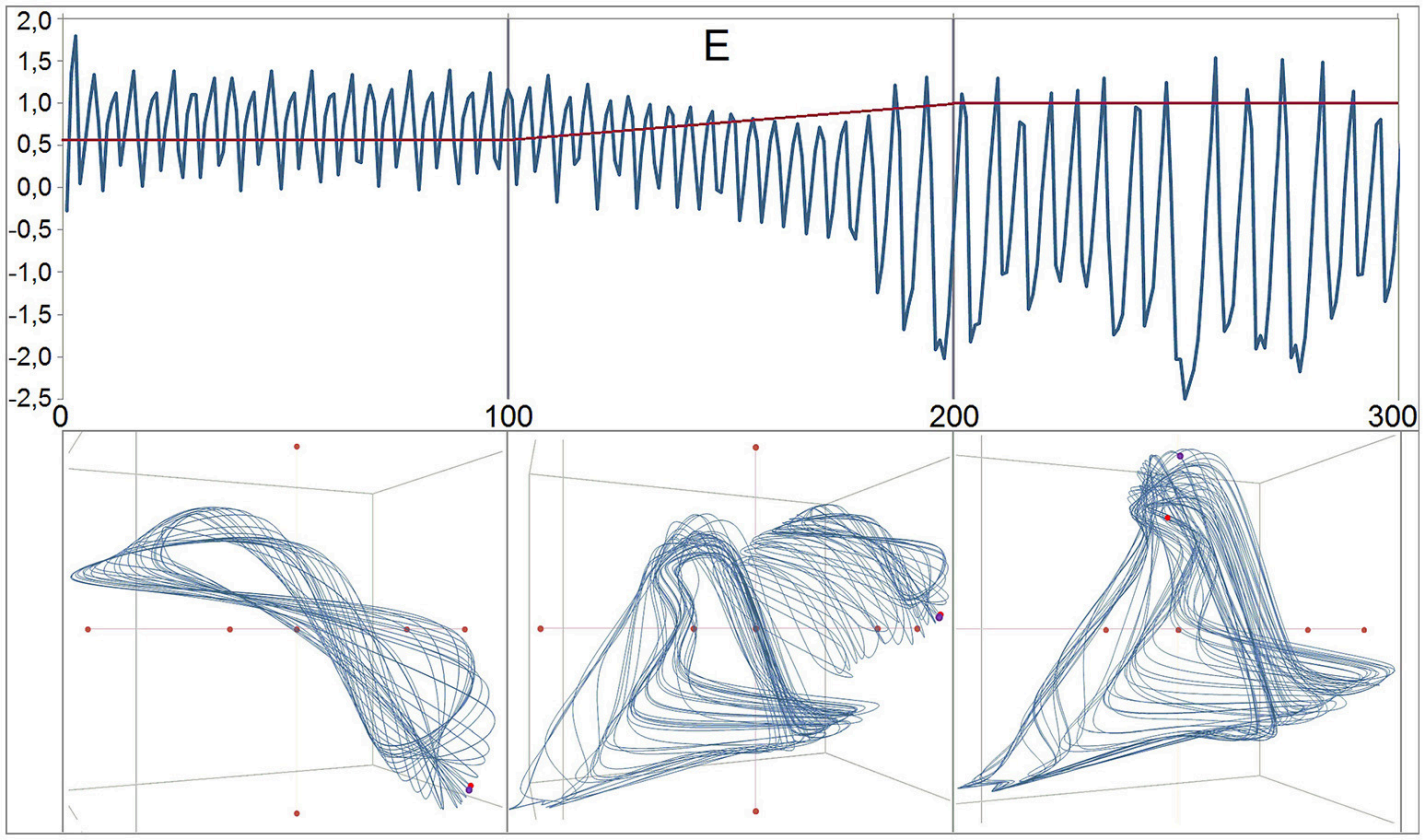
**Phase transition in the dynamics of the variable E**. The numbers at the y-axis refer to the values of E and the parameter *c*. The transition of the pattern depends on a stepwise linear increase of the parameter *c* from 0.60 to 1.00 between iteration 100 and 200. From iteration 0–100 the parameter is kept constant at 0.60 creating a certain dynamic pattern (attractor), after the 200st iteration *c* is constant at 1.00, producing another pattern at a lower mean level, at a lower frequency, and with higher amplitudes of the chaotic oscillations. The attractors are shown below the time series. For the generation of the attractors the discrete iterations were splined by the Excel standard spline function. During the linear stepwise increase of the control parameter, the transient attractor combines features of the pre and the post-attractor and by this is more complex than each of both.

The most evident and sustainable effects on the dynamic patterns of a system are due to the shift of its parameter(s). Like in classical physical Synergetics, it is the control parameter that changes the dynamics of the order parameters (Haken, 2004), what is called a *phase transition*. The effect of a parameter shift in *c* is demonstrated in Figure 11. A continuous parameter shift (continuous stepwise increase) in the sensitive range of the parameter can produce a discontinuous jump of the system dynamics (order to order transition, Haken and Schiepek, 2010).

## Discussion

This model and its dynamics illustrate that the assumptions and findings from common factors research and from related psychological topics (e.g., motivation, emotion regulation, information processing, and attachment) can be integrated into a comprehensive theory of change. This theoretical view takes seriously the notion that any conceptualization of psychotherapy should explain process and not only outcomes. Corresponding to empirical findings in psychotherapy research, the model is capable of producing chaotic dynamics, phase transition like phenomena, bi- or multi-stability, and phase transitions in response to parameter shifts. These are some of the most common dynamical features of therapeutic change processes observed in prior research. Therefore, the model may be seen as a first step toward a dynamic systems theory of psychotherapy, as well as a contribution to computational systems psychology.

One distinctive feature of the current approach compared to that of Liebovitch et al. (2011; Peluso et al., 2012), which focused on the co-evolution of emotional valences expressed by a therapist and his client, is that the current approach focuses on the psychological processes of clients in relation to their own experiences. The differential equations which were defined by the Libovich et al. group consist of segments of linear functions each defining the gradient of emotional changes which the client exerts on the therapist and vice versa. This leads to the prediction of stable fix-point attractors of the therapeutic relationship at the intercept of the valence functions, or to drop-outs, depending on the initial conditions in the two-dimensional phase portrait. Chaos is not possible within the scope of this model. Other actual mathematical models focus on dynamics of diseases, but not on psychotherapy. For example, Demic and Cheng (2014) reproduced different disease states of depression (depressive episode, recovery, relapse, remission) by a noise-driven dynamic systems model of one state variable. Huber et al. (2004) developed a nonlinear stochastic model of recurrent affective disorders. A mechanistic framework of brain network dynamics (Ramirez-Mahaluf et al., 2017) describes how abnormal glutamate and serotonin metabolisms mediate the interaction of vACC and dlPFC to explain cognitive and affective MDD symptoms and medical treatment effects (SSRI). Borsboom and Cramer (2013) and Wichers et al. (2015) model and analyze the features of cognitive and affective networks and their readiness to create psychopathological structures and dynamics. Previous simulation approaches used coupled nonlinear difference equations to understand the mechanisms and to reproduce the long-term evolutionary patterns of schizophrenia (Schiepek et al., 1992b). The current model adds to this body of computational approaches to understanding psychopathology and psychotherapy processes, providing a step toward a general theory at the intersection of each topic that is capable of producing each of the most relevant hallmarks of chaotic behavior and phase transitions.

### Epistemological remarks

Although the model as it is presented in this paper is based on our best empirically founded knowledge, it cannot be excluded that alternative conjectures and hypotheses concerning the relations and functions of the model will also be plausible or empirically grounded. One example may be the hypothesis motivation not only increases insight, but also that insight creates motivation. A better understanding of the psychological mechanisms of one's own problems can be encouraging, and may motivate further change. Additionally, emotions perhaps are not really necessary to create insight. Perhaps creative work like idiographic system modeling (Schiepek et al., 2015, 2016c) even is impeded by intensely experienced emotions, and illuminating insight may trigger positive instead of negative emotions. This encouraging insight concept may be called “Heureka model” and can be contrasted with the “look into the abyss” concept we adopted here. We decided as a first step to use the classical “look in the abyss” model, because it follows the conceptualizations of such recognized psychotherapy researchers as Grawe (2004), Greenberg (2002), Horowitz (1987), Mergentaler (1996), or Silberschatz (2009). Historically, this concept has been modified by modern psychologists but still is motivated by old psychoanalytic concepts of suppressed conflicts. Whatever explanation is preferred, one of the benefits of computer simulations is that you may implement and test both concepts (“experimentum *in silico*“). It is a matter of one's preference in the model-building phase, but then finally one must examine the degree of fit to data in the model testing phase.

Another criticism may concern the specification of parameter values to create chaotic dynamics. Indeed, the range of the parameters was restricted for creating the bifurcation diagrams (Figure 6)—a procedure which is called “windowing”—and put to specific values for creating other diagrams. We know this from other well-known bifurcation diagrams like the famous “Feigenbaum scenario” where in a range from 0 to 4 of the control parameter the first bifurcation appears at 3 and the chaotic dynamics at >3.5 (Feigenbaum, 1983; Strunk and Schiepek, 2006). Generally, nonlinear systems are able to produce chaotic dynamics, but its emergence as well as the “Gestalt” of the attractors depend on the fine tuning of specific parameter values (e.g., see the examples in Feigenbaum, 1983; Wolf et al., 1985; Schuster, 1989). Beyond mathematical models, the fine tuning of many parameters and natural constants in physics and biology for creating the world as we know it (from cosmology to the life of human beings) is a very universal phenomenon and an important topic in philosophy of nature—it is called the “anthropic principle” (Barrow and Tipler, 1988). The problem of fine tuning of parameters is fundamental, but in our case it is at least open to empirical verification if we are able to measure the parameters in each individual (see below).

The functions of the model are defined by specific shapes relating two or more constructs. This is necessary in order to concretize the nonlinear relations in terms of mathematical functions. Nevertheless, this does, to some extent, go beyond prior empirical findings. In many studies, findings are based on linear correlations or statistical testing of group differences. Here we defined psychological hypotheses as one would define physical laws. The defined relationships within the model are well justified, but realistically lack the same kind of rigor as physical laws. Thereby the functions idealize what we can know theoretically, while the field waits for future empirical specification and detailed definitions of psychological hypotheses. The functions as we defined them are by no means arbitrary, nor are they intentionally designed to create chaos. They were developed based on the most relevant knowledge in psychology and psychotherapy research (top-down), not by the dynamics they would produce or the search for optimally fitting functions (bottom-up).

The basic assumptions of our approach concern the nonlinearity of psychological mechanisms and the empirical findings on chaoticity and self-organization of psychotherapeutic change. This is why we used nonlinear dynamic systems theory, and in particular Synergetics, as the paradigmatic frame of this work. In this context, the distinction between order and control parameters plays an important role. The criteria for this differentiation is the reference to different time scales, the effects of control parameters on the shape of the functions interrelating the order parameters, and the knowledge of the systems under consideration (see Haken and Schiepek, 2010, for further clarification on this important topic).

### Limitations and further developments

One of the design decisions behind our model is, of course, the choice of a discrete time. It is well known that such choices can have strong effects on the resulting dynamics (Hütt, 2007; Strogatz, 2014). Finite-difference equations (or “maps”) can show deterministic chaos already in dimensions smaller than three, as opposed to continuous-time models based on ordinary differential equations. The most prominent example of a one-dimensional map with chaotic dynamics is certainly the logistic map (May, 1976), but also the Kaplan-Yorke map, the tent map, or the Hénon map (Collet and Eckmann, 2009).

Therefore, it is an open question (and will require further investigation), whether a continuous-time model based on similarly plausible assumptions about the nonlinear relationships between the dynamical variables will also have a chaotic regime. It should be noted, however, that the dimension of the model (*D* = 5) would in principle allow for chaoticity also in continuous time. For the present investigation we decided to explore the discrete-time version of the model. Our argument here is that the dynamical variables indeed only exist at discrete time points. The process of filling out the TPQ on a daily basis, in our view, goes along with a process of inspection within the clients, where formally the client maps his/her complex emotional pattern to the standardized variables contained in the TPQ. In this sense, the measurement process, induced by the TPQ, forms these variables only at discrete times.

Formally, we can consider the psychotherapy dynamics (at least for the phase space given by the 5 dynamical variables discussed here) as a system periodically driven by the TPQ. It is well-known that such periodic driving can trigger a complex dynamical response (e.g., Glass, 2001, for a general discussion and Hütt et al., 2002, for an empirical example of a temperature-driven photosynthetic activity of a plant leaf). While we believe that the psychotherapy itself is not affected by such a driver, the dynamical variables extracted by the TPQ certainly may justify our choice of a discrete-time model.

Our model still contains a large number of parameters shaping the various influence functions such that they conform to a wide range of empirical knowledge about psychotherapy. In the long run, a more minimal model, capable of reproducing the main ‘stylized facts’ (in the sense of Buchanan, 2012) should be constructed. Such a minimal model could support the view adopted here, that chaotic behavior is indeed an unavoidable consequence of the nonlinear interactions among the 5 dynamical variables. Understanding more deeply which model elements are necessary and sufficient for a particular dynamical behavior (e.g., chaotic dynamics) is a highly nontrivial task (e.g., Yordanov et al., 2011, where such an investigation has been performed for the model from Brandman et al., 2005).

An empirical test of the completed realistic model should assess: the parameter levels of *a, c, m*, and *r* of a client, the daily input on E, I, M, P, and S as experienced by the client, the initial conditions of the variables at the beginning of the therapeutic process, and the concrete dynamics of the variables. This should be possible since the parameters are widely used psychological constructs which can be assessed by known questionnaires, and the variables of the model correspond to 5 factors of the TPQ (see Haken and Schiepek, 2010) which is administered once per day in routine practice. The administration of the questionnaires is realized by an internet-based device, the Synergetic Navigation System (Schiepek et al., 2015, 2016a,c). This study also should contribute to a better understanding of the interindividual differences of dynamic patterns corresponding to the parameters which refer to the individual dispositions (traits) of the clients.

Of course, an extended concurrent validity study on the TPQ should be carried out. This is actually a work in progress, which is based on about 1,000 valid cases with almost complete process and outcome data (time series data <3% missings). These data are mined in routine practice of real-time monitoring in 5 Austrian and German hospitals (inpatient psychotherapy) and will be used for a further explorative and conformative factor analysis of the TPQ in order to confirm and to better understand the factors which correspond to the constructs of this model.

The aim of this contribution was to illustrate some basic features of human change dynamics. Further steps toward a more realistic model should include the following: (1) The parameters of the model not only determine the dynamics by shaping the functions, but are shaped themselves by the states and the dynamics of the system. In psychological terms, traits influence states and state dynamics; but the reverse is also true in that states (i.e., concrete experiences, cognitions, emotions, and behavior) may generate the competencies and the dispositions (traits) of an individual. This is the essential process of personality development and is explicitly intended by most psychotherapy approaches. In mathematical terms, the model has to be extended using equations that describe the parameter drift at a slower time scale than the state dynamics of the variables. This is an important extension, because as humans we cannot turn on the control parameters of dispositions or traits. We can only indirectly influence traits over time through experiences, cognitions, and behavior. This makes even more necessary a concept describing how experiences (in this model: variables) can change dispositions (parameters). (2) Future work on this model should incorporate experiences in everyday life and fluctuations from the inside of a system. This may be implemented using dynamic noise, which is processed by the network mechanism (Hütt, 2001). (3) Measurement noise results from poor reliability and accuracy of the assessment procedure, and will need to be considered in future work. (4) The input onto the system results from intended and planned interventions by the therapist or the therapeutic environment (in case of inpatient treatment). Also unscheduled and not intended experiences (e.g., in the social network of a client) can be experienced as therapeutic input.

### Practical and theoretical consequences

The consequences of a nonlinear conceptualization of psychotherapy, including the chaoticity of the dynamics, go far beyond theoretical reasoning (compare the Introduction section). Given the limited prediction horizon and the pronounced individuality of chaotic trajectories, manuals as guidelines for good practice are ruled out. Instead of dictating what has to be done by what steps in which session, the procedure has to be sensitive to the actual state of the dynamics, e.g., to its stability or instability. In other words, psychotherapy has to be client-centered in a dynamical sense. Indeed, when one examines empirical findings, the impact of manuals and manual adherence on therapy outcome is marginal (Webb et al., 2010; Wampold, 2015). Rather than predefined procedures, the role of real-time monitoring systems becomes significant, particularly if such systems not only assess and visualize the process, but also analyze its nonlinear features like dynamic complexity, pattern transitions, (in-)stability, or switching synchronization patterns (Schiepek et al., 2015, 2016a). The training of psychotherapists should communicate how to handle such systems (e.g., the Synergetic Navigation System) and how to use the results in a client-centered manner (continuous cooperative process control).

The model we outlined in this article supports the conceptualization of psychotherapy as encouraging and coaching the self-organizing processes of the client. Within this frame, interventions take different roles. First, they include all actions to realize the generic principles of psychotherapy (Schiepek et al., 2015). Second, interventions are the actions a therapist can arrange with the aim that the experiences of his client (in other words: the states of his variables E, I, M, P, and S) contribute to an improvement of the client's parameters, which in psychological terms correspond to dispositions or competencies. The way to change dispositions is by concrete experiences and behavior, because a direct modification of parameters (personality traits) seems to be impossible. Third, upon the backdrop of bi- or multistability within the client's psychological system, interventions may be viewed quite differently. Rather than mechanistic forces of invariant change, interventions are more like experimental inputs to explore the switching points, or to identify the triggers, which may turn on another attractor within the range of unique dynamic patterns of the system. In the metaphor of potential landscapes, the ball (the realized system behavior) is driven beyond the separatrix into another valley of the landscape—if it exists. The problem of this concept of interventions is that it should not be like poking around in the dark, but rather in close cooperation with the client, guided by mutual curiosity - a guided exploration of the capabilities of each client's unique personality.

Finally, the model is one piece of a larger puzzle toward an integrative conceptualization of psychotherapy. Besides a general theoretical framework or scientific paradigm, it needs for a concrete theory of change dynamics. This will allow for an optimizion of our understanding of the mechanisms of therapy in general, and in the particular case of each client, given that clients unique dispositions and initial conditions. There are numerous other pieces of the larger puzzle, such as array of available intervention tools. This might be the eclectic part of the whole with different psychotherapy schools as contributors to an intervention pool. A method of case formulation is needed, combining different perspectives and particular hypotheses into a systemic network model (Schiepek et al., 2016c). Theory-based heuristics will be important for the micro-decisions during the ongoing process of a continuous cooperative process control (generic principles, Schiepek et al., 2015). Similarly methods for therapy monitoring and therapy feedback, as it is given by the Synergetic Navigation System, along with outcome and process evaluation integrated into the routine practice of inpatient and outpatient psychotherapy will improve the field. Necessary for ongoing science and training will be the development of an idea of how to bridge the gap between practice and research, and how to use clinical practice as a research field. Finally an elaborated concept of the competencies a scientist-practitioner should be made available if he/she wants to understand, analyze, and manage complex, nonlinear, and self-organizing human systems (systems competence). Computer-based simulations as presented in this article can take a role in the training of how to manage therapies in complex, chaotic, and partially nontransparent systems (Mainzer, 2007; www.psysim.de by Schöller and Schiepek).

## Dedication

This article is dedicated to Prof. Dr. Dr. h.c. mult. Hermann Haken, the founder of Synergetics, to his 90th birthday.

## Author contributions

GS designed the psychological model of psychotherapeutic change dynamics, contributed to the mathematical formalization, and wrote the paper. KV and GS realized the mathematical formalization of the model. WA supported the realization of the project and prepares the empirical validation of the model. MH supervised the mathematical procedures and contributed important advices to the optimization of the project. KS contributed to the psychological underpinning of the model (e.g., by screening empirical findings) and prepares its empirical validation. DP provided edits and text to clarify use of English Language, grammar and style, as well as content expertise on dynamical systems concepts and psychotherapy. HS contributed to the mathematical formalization, performed the numerical simulations and produced the figures.

### Conflict of interest statement

The authors declare that the research was conducted in the absence of any commercial or financial relationships that could be construed as a potential conflict of interest.

## Footnotes

^1^In a short side note, it should be said that chaos is different from critical instability. Chaos is a complex dynamic pattern (unpredictable yet ordered, not disordered *per se*), which is represented by its (*strange* or *chaotic*) *attractor*. To be identified as chaotic, a certain stability of this pattern is required, but of course, it can change to another chaotic attractor (i.e., *chaoto-chaotic* phase transition). In contrast to this, *critical instability* is typically considered to be a transient phenomen, which endures only for a relatively short period of time. As a precursor of dynamic transitions it is, at least in some aspects, a marker of disorder or noise.

## Appendix

$$\begin{array}{l} \text{E}\left(\text{E},\text{I},\text{P},\text{S},\text{c},\text{r},\text{m}\right)\;=\;\frac{1}{{1\;+\text{ e}}^{-10\text{ E}}}\;-\;c\;+\;\frac{1}{{1+\text{ e}}^{-20\text{ I }\cdot \;\left(1\;-\;\frac{\text{c }+\text{ r}}{2}\right)\;+\;5}}\;+\\ +\;\frac{\frac{-1}{1\;+\;{\text{e}}^{\left(2\;+\;3\;\cdot \;\left(1\;-\;\frac{\text{c }+\text{ m}}{2}\right)\right)\;\cdot \;\text{P}}}\;+\;0,5\;+\;0,5\;\cdot \;\left(1\;-\;\frac{\text{c }+\text{ m}}{2}\right)}{1\;+\;{\text{e}}^{\;25\;\cdot \;\left(1\;-\;\frac{\text{c }+\text{ m}}{2}\right)\;\cdot \;\left(\text{P}\;-\;0,2\;-\;0,75\;\cdot \;\left(1\;-\;\frac{\text{c }+\text{ m}}{2}\right)\right)}}\;+\\ +\;\frac{1,25}{1\;+\;{\text{e}}^{5\text{S}\;-\;0,5}}\;-\;0,5\;-\;0,5\text{ m} \end{array}$$

$$\begin{array}{l} \text{I}\left(\text{E},\text{M},\text{S},\text{a},\text{c}\right)\;=\;\frac{1}{{1\;+\text{ e}}^{-20\text{ E }\cdot \;\left(\frac{\text{a }+\text{ c}}{2}\right)\;+\;5}}\;+\;\frac{1}{{1\;+\text{ e}}^{-20\text{ M }\cdot \;\left(\frac{\text{a }+\text{ c}}{2}\right)\;+\;5}}\;+\;\frac{1}{{1\;+\text{ e}}^{-20\;\cdot \;|\text{ S }|\;\cdot \text{ c }+\;5}} \end{array}$$

$$\begin{array}{l} \text{M}\left(\text{P},\text{S},\text{r},\text{m}\right)=\frac{1,261}{\left(1\;+\;{\text{e}}^{\left(\text{P}\;-\;0,05\;-\;0,85\text{ m}\right)\;\cdot \;\left(10,1\;+\;19,9\text{ m}\right)}\right)\;\cdot \;\left(1\;+\;{\text{e}}^{-\left(\text{P}\;-\;0,43\;+\;0,03\text{ m}\right)\;\cdot \;\left(7\;-\;3\text{ m}\right)}\right)}\\ \;+\frac{-1}{1\;+\;{\text{e}}^{5\;\text{S}}}+\frac{\text{r }+\text{ m}}{2} \end{array}$$

$$\begin{array}{l} \text{P}\left(\text{E},\text{S},\text{c},\text{r}\right)=\frac{1}{{1\;+\text{ e}}^{-10\text{ E}}}\text{ c}+\frac{1,2}{1\;+{\text{ e}}^{5\;\text{S}-\;0,5}}\;-\;0,2\;-\;0,8\text{ r} \end{array}$$

$$\begin{array}{l} \text{S}\left(\text{E},\text{I},\text{M},\text{P},\text{S},\text{a},\text{c},\text{m},\text{r}\right)=\frac{1,3}{1\;+\;{\text{e}}^{5\text{ E }-\;0,5}}\;-\;0,65+0,35\;\cdot \;\left(\text{c }+\text{ m }-\;1\right)+\frac{1}{{1\;+\text{ e}}^{-20\text{ I }\cdot \;\frac{\left(\text{a }+\text{ m }+\text{ r}\right)}{3\;}\;+\;5}}+\\ \;+\frac{1}{{1+\text{ e}}^{-20\text{ M }\cdot \;\frac{\left(\text{a }+\text{ m }+\text{ r}\right)}{3}\;+\;5}}\;-\;\frac{1}{{1+\text{ e}}^{20\text{ M }\cdot \;\left(1\;-\;\frac{\left(\text{a }+\text{ m }+\text{ r}\right)}{3}\right)\;+\;5}}+\\ \;+\frac{1,25}{1\;+\;{\text{e}}^{5\text{ P }-\;0,5}}\;-\;0,5\;-\;0,5\;\cdot \;\left(1\;-\;\frac{\text{c }+\text{ m}}{2}\right)+\frac{1}{{1+\text{e}}^{-10\text{ S}}}\;+\;\frac{\text{m}+\text{r}}{2}\;-\;1 \end{array}$$
